# Common and Strain-Specific Post-Translational Modifications of the Potyvirus *Plum pox virus* Coat Protein in Different Hosts

**DOI:** 10.3390/v12030308

**Published:** 2020-03-12

**Authors:** Marta Hervás, Sergio Ciordia, Rosana Navajas, Juan Antonio García, Sandra Martínez-Turiño

**Affiliations:** 1 Department of Plant Molecular Genetics, Centro Nacional de Biotecnología (CNB-CSIC), Campus Universidad Autónoma de Madrid, 28049 Madrid, Spain; mhervas@cnb.csic.es; 2 Proteomics Unit, Centro Nacional de Biotecnología (CNB-CSIC), ProteoRed ISCIII, 28049 Madrid, Spain; sciordia@cnb.csic.es (S.C.); rnavajas@cnb.csic.es (R.N.)

**Keywords:** capsid protein, *O*-GlcNAcylation, phosphorylation, sharka, potyvirus

## Abstract

Phosphorylation and *O*-GlcNAcylation are widespread post-translational modifications (PTMs), often sharing protein targets. Numerous studies have reported the phosphorylation of plant viral proteins. In plants, research on *O*-GlcNAcylation lags behind that of other eukaryotes, and information about *O*-GlcNAcylated plant viral proteins is extremely scarce. The potyvirus *Plum pox virus* (PPV) causes sharka disease in *Prunus* trees and also infects a wide range of experimental hosts. Capsid protein (CP) from virions of PPV-R isolate purified from herbaceous plants can be extensively modified by *O*-GlcNAcylation and phosphorylation. In this study, a combination of proteomics and biochemical approaches was employed to broaden knowledge of PPV CP PTMs. CP proved to be modified regardless of whether or not it was assembled into mature particles. PTMs of CP occurred in the natural host *Prunus persica*, similarly to what happens in herbaceous plants. Additionally, we observed that *O*-GlcNAcylation and phosphorylation were general features of different PPV strains, suggesting that these modifications contribute to general strategies deployed during plant-virus interactions. Interestingly, phosphorylation at a casein kinase II motif conserved among potyviral CPs exhibited strain specificity in PPV; however, it did not display the critical role attributed to the same modification in the CP of another potyvirus, *Potato virus A*.

## 1. Introduction

Numerous viral proteins can be post-translationally modified at different stages of infection, probably a result of co-evolutionary strategies, whereby viruses acquire the ability to hijack host enzymes [1]. There are abundant examples of proteins of animal viruses that are targeted by phosphorylation [2,3,4,5], one the most widespread post-translational modifications (PTMs), which dynamically regulate functions of a large number of cellular proteins. Many viral products of viruses infecting plants, both non-structural [6,7,8,9,10,11,12] and structural [13,14,15,16,17] proteins, have also been found to be modified by phosphorylation.

The genus *Potyvirus* is one of the largest and more economically relevant groups of plant viruses [18]. Potyviruses have an approximately 10 kb single-stranded linear positive-sense RNA genome, which is encapsidated in flexuous rod particles formed by multiple subunits of a single capsid protein (CP). The main open reading frame (ORF) of potyviral genomic RNA is translated into a large polyprotein, which includes the CP at its C-end, and it is proteolytically processed by three virus-encoded proteinases [19,20]. A frameshift to an internal small ORF as a result of RNA polymerase slippage permits the synthesis of an additional transframe product [21,22,23]. Phosphorylation affecting CP of the potyvirus *Potato virus A* (PVA) has been thoroughly studied. PVA CP is phosphorylated at the C terminus of the protein by casein kinase II (CK2) in the motif (S/T)XX(D/E) conserved in most potyviruses [24,25,26]. It has been suggested that the existence of a delicate balance between PVA CP phosphorylation-dephosphorylation would facilitate the controlled RNA translation/replication switching required for a successful viral infection [26,27]. The CP of another potyvirus, *Plum pox virus* (PPV), is also phosphorylated [28,29]. Interestingly, PPV CP is also modified by a second PTM, *O*-GlcNAcylation [28,30], which is known to be functionally linked to phosphorylation in many dynamic regulatory activities [31] and has been shown to play important roles in animal virus infections [32,33,34,35]. This makes PPV CP an attractive subject to study the joint contribution of these closely related PTMs in the regulation of viral infection.

CP from virions of the PPV R isolate, which belongs to strain D, has been shown to be extensively *O*-GlcNAcylated by the *O*-GlcNAc transferase (OGT) SECRET AGENT (SEC) along the N-terminal region of the protein [30,36,37]. PPV CP also displays a complex phosphorylation pattern, which spans both N terminus and the core region of the protein [38,39]. Neither *O*-GlcNAcylation nor phosphorylation of CP is essential for virus viability, but both appear to contribute to infection efficiency [30,37,38]. It has been proposed that dynamic phosphorylation and *O*-GlcNAcylation at N terminus of the protein regulate CP stability, thus providing appropriate CP amounts for each phase of viral infection, while phosphorylation at core region controls viral particle assembly/disassembly [39].

PPV naturally infects *Prunus* trees causing sharka, the most devastating viral disease that affects stone fruit trees worldwide, but it is also able to infect a wide range of experimental herbaceous hosts, among them *Nicotiana* spp. [40,41]. All studies about *O*-GlcNAcylation and phosphorylation affecting PPV CP have been narrowed to infections in herbaceous hosts, mainly in *Nicotiana* spp., and no data concerning PTMs of CP during infections in natural woody hosts are available. On the other hand, up to 10 different PPV strains have been described [42,43]. Although earlier assays, done with isolates belonging to strains D, M, and Rec, suggested that *O*-GlcNAcylation and phosphorylation affecting CP may be a common attribute of PPV species [28,29,44], thorough proteomic analyses of PTMs have only been conducted for the CP of the PPV R isolate, belonging to strain D.

In this work, we used a combination of proteomics and biochemical approaches to study *O*-GlcNAcylation and phosphorylation of PPV R when infecting seedlings of the natural host of the pathogen *Prunus persica*, as well as PTMs of PPV CP from isolates of strains Rec and C in *Nicotiana* plants. Our results indicated that modification of CP by *O*-GlcNAcylation and phosphorylation took place in at least two evolutionarily distant hosts, *Nicotiana* and *Prunus*, and was a general feature of different PPV strains. Moreover, we showed that *O*-GlcNAcylation and phosphorylation were not exclusive to CP arranged in virions. Remarkably, although phosphorylation at conserved casein kinase II (CK2) motif of CP did not have in PPV the same pivotal role reported for PVA, it did appear to have some strain-specific contribution to PPV infection.

## 2. Materials and Methods

### 2.1. Viral cDNA Clones and other Plasmids

Plasmid pSN-PPV-5′BD-GFP (Pasin F. and García J.A., unpublished results [45]) was used to infect both *P. persica* and *Nicotiana benthamiana* plants by agroinfiltration. This plasmid was created by engineering the chimeric cDNA of PPV pICPPV-5′BD-GFP [46], also used to infect *Prunus* plants but by biolistic method, into pSN-PPV [47] (Figure S1).

The following plasmids were used to inoculate *N. benthamiana* plants by hand rubbing: a GFP-tagged full-length cDNA clone of PPV-R, pICPPV-NK-lGFP, and a chimeric clone pICPPV-CPSwCM-R, which includes in the backbone of PPV-R [48] the CP coding sequence of PPV-SwCM, an isolate belonging to strain C.

Point mutations affecting alleged phospho-targets threonine 304 (T304) in the CP of PPV-R and threonine 306 (T306) in that of SwCM were respectively engineered into plasmids pICPPV-NK-lGFP and pICPPV-CPSwCM-R (Figure S1). Replacements of ACA codon by GCA or GAT were chosen to, respectively, achieve changes of threonine to alanine or aspartic acid with as few changes as possible in the RNA sequence. The control mutation changing to asparagine (AAT) was conceived to get a maximum distortion in RNA folding.

Mutations at T304 and T306 were created by site-directed mutagenesis using a three-step PCR approach, as previously described [49]. Primers and templates used in each of the amplification are listed in Tables S1 and S2. Fragments containing point mutations in triplet coding T304 were digested with SacI and XbaI and inserted back into plasmid pICPPV-NK-lGFP to obtain final constructs R-T304A, R-T304D, and R-T304N. To obtain constructs SwCM-T306A, SwCM-T306D, and SwCM-T306N, fragments mutated in triplet coding T306 were inserted back into plasmid pICPPV-CPSwCM-R, after digestion with SpeI and XbaI (Figure S1).

### 2.2. Plant Growth Conditions and Viral Inoculation

Plants were cultured in a glasshouse at 19–23 °C and a 16 h/8 h (light/dark) photoperiod, except for *P. persica* and *N. benthamiana* plants agroinoculated with pSN-PPV-5′BD-GFP, which were grown in a climate chamber at 22 °C with the same photoperiod. For agroinoculation, young *P. persica* cv. GF305 and *N. benthamiana* plants (four-to-six-leaf stage) were infiltrated with cultures of *Agrobacterium tumefaciens* GV3101 (pMP90, pJIC SA_Rep) transformed with plasmid pSN-PPV-5′BD-GFP, as previously described [50]. In the case of *Prunus* plants, the agrobacterium pellet was suspended in inoculation buffer to reach an OD_600_ of 1, and leaves were infiltrated by pressing strongly and repeatedly on the syringe plunger, in overlapping patches, to cover most of the foliar area.

For manual inoculation of *N. benthamiana* plants, pICPPV-NK-lGFP- or pICPPV-CPSwCM-R-derived plasmids were dispensed in three leaves per plant (5 to 10 µL, at 1 µg/µL, per leaf) and rubbed using Carborundum as an abrasive agent. Similarly, crude homogenates from tissue infected with PPV-BOR-3, an isolate belonging to strain Rec, were used to inoculate *N. benthamiana* and *Nicotiana clevelandii* plants (three leaves per plant), by rubbing 10 µL of extract (2 mL of 5 mM sodium phosphate pH 7.2 per g of tissue) per leaf. Serial passages of PPV-NK-lGFP- and PPV-CPSwCM-R-derived mutants through *N. benthamiana* plants were also carried out by hand-rubbing with crude homogenates of infected leaves, as explained above.

Biolistic inoculation of *P. persica* cv. GF305 with microgold particles coated with DNA of pICPPV-5´BD-GFP was performed with a Helios gene gun device (Bio-Rad, Hercules, CA, USA), as previously described [37].

### 2.3. Assessment of Viral Infection and Virion Purification

Viral infection was monitored by visual inspection of symptoms and by observing GFP fluorescence under a long-wavelength UV lamp or a stereomicroscope, as previously described [38]. Viral accumulation was determined by Western blot analysis using an in house-produced anti-CP serum, as described by Perez et al. [37]. To analyze viral progenies, the complete CP coding sequence was amplified by reverse transcription and PCR, preceded by immunocapture (IC-RT-PCR) [37], using oligo 2429 as a reverse primer and forward primers SM13-lGFP (for PPV-NK-lGFP-derived mutants) or oligo 80 (for PPV-CPSwCM-R-derived mutants) (Table S2). Amplicons were subjected to Sanger sequencing by Macrogen Europe (Amsterdam, Netherlands).

Virions were purified by the method previously described in Laín et al. [51], slightly modified [30], or by a modification of the protocol developed by Sheveleva et al. [52], adjusted to improve yield and purity of samples, as follows. Systemically infected leaves were collected at 21–22 days post-inoculation (dpi), and frozen tissue was ground in liquid nitrogen for 10 min in an electric blender with an extraction buffer composed of 0.02 M HEPES, 0.2 M sucrose, and 10 mM sodium diethyldithiocarbamate, pH 6.8 (4 mL of buffer per g of fresh tissue). Homogenized extracts were clarified by low-speed centrifugation (15 min at 5465× *g*). The supernatant was filtered through a Miracloth membrane (Merck, KGaA, Darmstadt, Germany), and Triton X-100 was added slowly to a final concentration of 5%. The stirring process was maintained during 1 h, and then the mixture was ultra-centrifuged through a layer of 20% sucrose in 0.02 M HEPES buffer, pH 6.8 (210 min at 71,109× *g*). The resulting pellet was suspended overnight in 0.1 M sodium borate, 5 mM EDTA buffer, pH 8.2 (borate-EDTA buffer), with a magnetic stirrer. After removal of debris by centrifugation in a tabletop microfuge, the supernatant was subjected to a new step of ultracentrifugation through a continuous sucrose gradient (10%–40%) in a borate-EDTA buffer (50 min, at 71,109× *g*). Fractions in which virions are usually concentrated were diluted in the borate-EDTA buffer, adjusted to 0.005% of Triton X-100, and dialyzed during 3 h through a 10 kDa exclusion membrane *SnakeSkin Dialysis Tubing* (Thermo Scientific, Rockford, IL, USA) against borate-EDTA buffer with 0.005% Triton X-100. Dialyzed samples were centrifuged at 81,630× *g* for 180 min, and the pellet was resuspended for at least 2 h in 5 mM sodium borate buffer, pH 8.2 with a magnetic stirrer. All purification steps were carried out at 4 °C. The yield and quality of samples were checked by 12% SDS-PAGE. In cases when an excess of plant contaminants was detected, samples were subjected to an additional purification step, by repeating the sucrose gradient sedimentation and further concentration by ultracentrifugation.

### 2.4. Fractionation by Centrifugation and Immunoprecipitation of CP

Crude extracts from systemically infected *N. benthamiana* leaves, prepared as previously described [53], were subjected to centrifugation in 33 mL continuous sucrose gradients (10%–40%) for 2 h at 140,992× *g*. Aliquots were collected by gravity from the bottom with a capillary tube and analyzed by Western blot anti CP. For CP immunoprecipitation, pools of one of the fractions of 4 independent gradients (4 mL in total) were mixed with an equal volume of 200 mM Tris-HCl, 600 mM NaCl, 10 mM DTT, 0.4% Triton X-100 buffer, pH 7.4 (BI buffer) supplemented with 0.2 % cOmplete EDTA-free Protease Inhibitor Cocktail (Roche, Mannheim, Germany). Then, 10 µL of anti-CP rabbit polyclonal serum was added to the mixture. After five hours of incubation at 4 °C in rotary motion, 50 μL of Protein A Sepharose Fast Flow (GE Healthcare Life Sciences, Uppsala, Sweden) was added, and incubation continued overnight under the same conditions. Immuno-complexes were collected by centrifugation in a tabletop centrifuge and washed three times with BI buffer. Finally, immunoprecipitated CP was eluted from sepharose beads with 80 μL of disruption buffer (125 mM Tris–HCl pH 7.5, 2% SDS, 0.1% bromophenol blue, 6 M urea, 4% glycerol, and 5% β-mercaptoethanol).

### 2.5. Proteomic Approaches

#### 2.5.1. Matrix-assisted Laser Desorption Ionization–Time of Flight (MALDI-TOF)

PPV virions (1–5 µg) were digested with 50 ng (10 ng/µL) of Pierce MS grade trypsin (ThermoFisher, Rockford, IL, USA) in 50 mM ammonium bicarbonate for 20 min at approximately 22 °C, desalted with a Zip-Tip reverse-phase C18 column, and eluted in 70% aqueous acetonitrile and 0.1% trifluoroacetic acid (TFA), as previously described [30]. Eluates were dried by speed vacuum centrifugation and resuspended in 30% acetonitrile, 15% isopropanol, and 0.5% TFA. A 1-μL aliquot of each peptide mixture was deposited manually on a 384-well OptiTOF Plate (SCIEX, Framingham, MA, USA) and allowed to dry at room temperature. A 1-μL aliquot of matrix solution (10 mg/mL 2,5-dihydroxyacetophenone in 50% aqueous acetonitrile and 100 mM ammonium citrate) was then deposited onto the dried digests. After drying at room temperature, samples were analyzed in an ABi 4800 MALDI TOF/TOF mass spectrometer (SCIEX) in positive ion linear mode, as described previously [54]. The detection mass range was set between 1000 and 10,000 m/z.

#### 2.5.2. Liquid Chromatography-Tandem Mass Spectrometry (LC-MS/MS)

PPV virions were precipitated with methanol–chloroform mix (4:1) and reconstituted in 8 M urea, 25 mM ammonium bicarbonate solution. After following reduction (10 mM dithiothreitol) and alkylation (50 mM iodoacetamide) in 25 mM ammonium bicarbonate, samples were digested at 37 °C with Lys-C (6 h) and trypsin (15 h) at an enzyme:substrate ratio of 1:50. Phospho-peptide enrichment procedure concatenated two in-house packed microcolumns—Immobilized Metal Affinity Chromatography (IMAC) and Oligo R3 polymeric reversed-phase—, which provided selective purification and sample desalting prior to LC-MS/MS analysis [55]. Phospho-peptide-enriched mixtures were analyzed on a nano liquid chromatography coupled to MS/MS Triple TOF mass spectrometer, operated in data-dependent acquisition mode (selection and fragmentation of 15 precursor ions), as described in Martínez-Turiño et al. [38].

MS/MS spectra were exported to mgf format and searched using an in-house licensed Mascot Server 2.6.1 (http://www.matrixscience.com; Matrix Science, London, UK) against a PPV protein database, containing 13 protein sequences together with commonly occurring contaminants. In addition to Mascot, an in-house licensed Peaks Search engine 7.5 (http://www.bioinfor.com; BSI, Waterloo, Canada) was also used. Search engines considered fixed carbamidomethyl modification of cysteine and the following variable modifications: oxidation of methionine, phosphorylation and glycosylation of serine and threonine, and phosphorylation of tyrosine. No enzyme restriction was considered.

The mass spectrometry proteomics data have been deposited to the ProteomeXchange Consortium via the PRIDE [56] partner repository with the dataset identifier PXD017780 and 10.6019/PXD017780.

## 3. Results

### 3.1. O-GlcNAcylation and Phosphorylation of PPV CP also Affect Protein not Assembled in Mature Viral Particles

**Figure 1:**
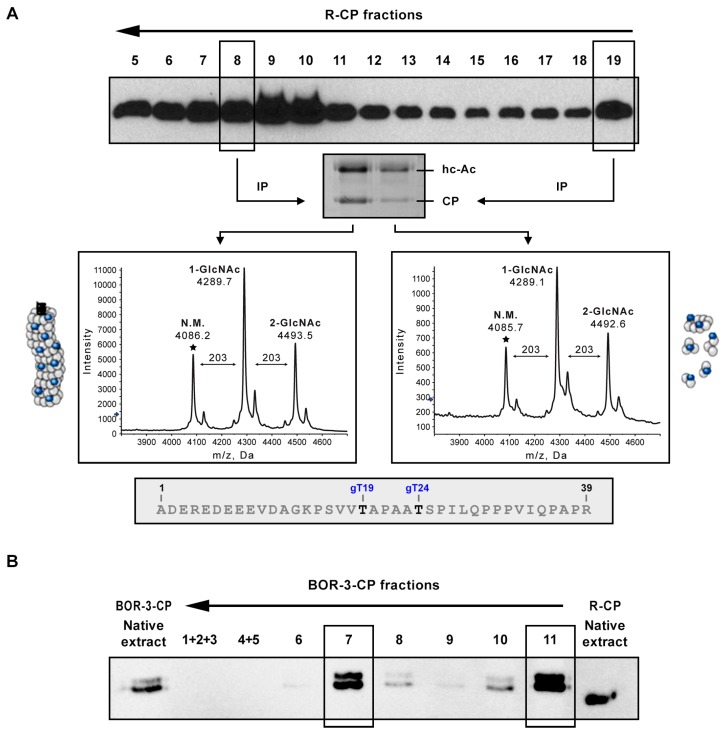
Analysis of post-translational modifications affecting the coat protein (CP) of the *Plum pox virus* (PPV) when assembled or unassembled in viral particles. Upper non-inoculated leaves of *Nicotiana benthamiana* plants infected with PPV-R (strain D) (**A**) or PPV-BOR-3 (strain Rec) (**B**) were collected at 17 days post-inoculation. Crude extracts from plant tissue were prepared under native conditions and subjected to sucrose gradient centrifugation. (**A**) Western blot anti-CP of sucrose gradient fractions from PPV-R-infected plants. Fractions containing small complexes and free CP (19) or viral particles (8) were separately immunoprecipitated (IP) with anti-CP serum. CP recovery following IP was checked by SDS-PAGE and Coomassie blue staining. Immunoprecipitated CP and immunoglobulin heavy chain are indicated. *O*-GlcNAcylation of the CP of PPV-R (R-CP) from each immunoprecipitated fraction was analyzed by matrix-assisted laser desorption/ionization-time flight. Spectra corresponding to peptide 1–39 are displayed. Mass/charge ratios (*m*/*z*) assigned to relevant peaks, as well as predicted number of *O*-GlcNAc residues modifying each peptide species, are shown. Stars mark unmodified parental (N.M.) peaks. The sequence of peptide 1–39 and their *O*-GlcNAcylated residues are shown below spectra. (**B**) Western blot anti-CP of sucrose gradient fractions from PPV-BOR-3-infected plants. Fractions containing small complexes and free CP (11) or virus-like particles (7) are boxed. The double band of the CP of PPV-BOR-3 (BOR-3-CP) indicates its partial phosphorylation. Non-fractioned extract (native extract) of PPV-BOR-3 and PPV-R, whose CP migrates as a single band, are also shown. Both in (**A**) and (**B**), the arrow on top of Western blots indicates the sedimentation sense.

Previously, we reported that during infections of herbaceous hosts, the capsid protein (CP) from the PPV-R isolate (R-CP) assembled in virions was extensively *O*-GlcNAcylated by the *O*-GlcNAcyltransferase (OGT) SEC [36,37] and phosphorylated [38]. To determine whether these PTMs also affect the CP that is free and/or is part of small complexes, PPV viral particles were separated from CP small aggregates and free protein. For this purpose, *N. benthamiana* plants were inoculated with a PPV-R cDNA clone, pSN-PPV, and systemically infected leaves were homogenized in a phosphate buffer. The resulting extract was fractionated by sucrose gradient centrifugation, and collected fractions were analyzed by Western blot anti-CP (Figure 1A).

A virion-containing fraction (fraction 8) and the gradient-top fraction, where small complexes and free CP are retained (fraction 19), were separately immunoprecipitated using a specific anti PPV CP serum. Both samples were trypsinized, and *O*-GlcNAcylation of peptide 1–39, which has been shown to contain up to two *O*-GlcNAcylations, at T19 and T24 [37], was analyzed by MALDI-TOF. *O*-GlcNAcylation patterns of CP assembled and non-assembled in virions ran similarly, and the intensity ratios among non-modified, mono-, and di-*O*-GlcNAcylated forms for both samples were comparable (Figure 1A).

CP of the PPV-BOR-3 isolate (BOR-3-CP) has the peculiarity of migrating as a doublet in SDS-PAGE, presumably because of its partial phosphorylation [29]. To assess whether this PTM depends on the level of CP assembly, a crude extract from leaves of *N. benthamiana* plants systemically infected with PPV-BOR-3 was fractionated by sucrose gradient centrifugation. Western blot analysis of all gradient fractions detected the double band that is characteristic of phosphorylated BOR-3-CP, both in virion-hosting fraction 7 and in fraction 11, containing CP not assembled in virus particles (Figure 1B). This result indicated that, as in the case of *O*-GlcNAcylation, CP phosphorylation was not restricted to fully formed viral particles but could also affect protein at lower assembly levels.

### 3.2. Post-Translational Modifications Affecting PPV CP Take Place in the PPV Natural Host Prunus Persica

PTMs of PPV CP had been shown to take place in experimental herbaceous hosts: *O*-GlcNAcylation in *Nicotiana* spp. and *Arabidopsis thaliana* [37] and phosphorylation in *Nicotiana* spp. [38,39]. However, until now, it was unknown whether these modifications also affect PPV CP in natural PPV woody hosts.

**Figure 2:**
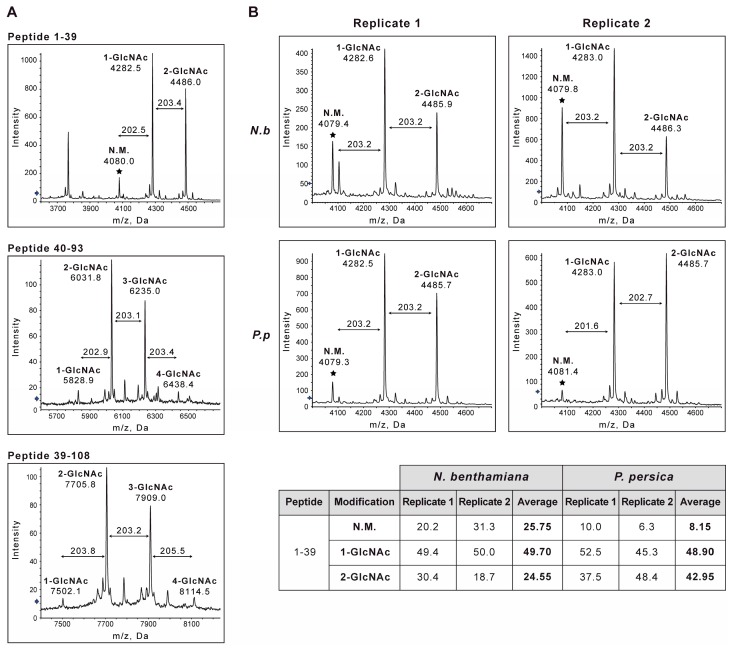
The *O*-GlcNAcylation pattern of the coat protein (CP) of *Plum pox virus* (PPV) in virions purified from *Prunus persica*. (**A**) Matrix-assisted laser desorption/ionization-time flight (MALDI-TOF) spectra showing peaks corresponding to different species of peptides 1–39, 40–93, and 39–108 of PPV CP. (**B**) Comparison of MALDI-TOF spectra showing peaks corresponding to different species of peptide 1–39 from PPV virions produced in *Nicotiana benthamiana* (*N.b*) and *P. persica* (*P.p*). The table shows relative accumulations of the species of peptide 1–39 non-modified (N.M.), mono-*O*-GlcNAcylated (1-GlcNAc), and di-*O*-GlcNAcylated (2-GlcNAc). *O*-GlcNAcylation levels were estimated from the peak intensities (*Y*-axis) and calculated by dividing each maximum signal intensity by the sum of those of all peptide species (average values of two biological replicas). In both (**A**) and (**B**), mass/charge ratios (*m*/*z*) assigned to relevant peaks, differences in mass among peaks attributed to *O*-GlcNAc groups, as well as predicted number of *O*-GlcNAc residues modifying each peptide species, are depicted. The asterisk marks the unmodified peak (N.M.) of each tryptic peptide.

To solve this question, *Prunus persica* seedlings were biolistically inoculated with the full-length chimeric clone pICPPV-5´BD-GFP, which expresses R-CP. Infection was verified by visual inspection for symptoms (light chlorotic or yellow spots, yellow line patterns along veins and leaf distortion) and by monitoring of virus-derived GFP fluorescence. Virions were purified from systemically infected tissue, partially digested with trypsin, and subjected to MALDI-TOF analysis to inquire about the putative *O*-GlcNAcylation of CP. The N-terminal tryptic peptide 1–39 of CP was detected as both non-glycosylated and mono- and di-*O*-GlcNAcylated forms (Figure 2A). In addition, spectra revealed peaks matching with a form of peptide 40–93 that house up to 4 *O*-GlcNAc residues (Figure 2A). *O*-GlcNAcylations that modify peptide 40–93 were redundantly found in peptide 39–108 (Figure 2A).

These *O*-GlcNAcylation patterns fit with the existence of *O*-GlcNAcylated residues previously identified in R-CP produced in infections of *Nicotiana* spp. plants (T19, T24, T41, T50, T53, T54, and/or T58, and S65) [37,38]. However, the level of *O*-GlcNAcylation at these sites appeared to be somewhat higher in this experiment compared to that found in previous works. To confirm such a difference and assess whether it is caused by the use of the PPV-5′BD chimera or it is specifically linked to the host type, *P. persica* and *N. benthamiana* plants were inoculated with pSN-PPV-5′BD-GFP. This last plasmid is another PPV-5′BD-GFP chimeric clone but allowing inoculation of PPV by agroinfiltration. After agroinoculation, the *O*-GlcNAcylation pattern of peptide 1–39 was analyzed in two biological samples of virions purified from each of the infected host types.

Estimates based on peak intensities showed a relative abundance of the non-modified peptide more than three times higher in *N. benthamina* samples compared with those obtained from *P. persica* (average of 25.8% vs. 8.2%). Accordingly, the amount of di-*O*-GlcNAcylated peptide was almost twice in the *Prunus* sample compared to that of *N. benthamina* (average of 43.0% vs. 24.6%) (Figure 2B).

Although the sample size of this study was not large enough to perform a statistical analysis that would rigorously support the results, our data suggested that *O*-GlcNAcylation of R-CP was enhanced in the natural PPV host compared with that occurring in herbaceous plants.

Former results had shown that R-CP was targeted by phosphorylation in PPV-infected *N. benthamiana* at specific serines (S25, S81, S101, and S118) and, more sporadically, at threonines (T71, T106, T254, and T313) [38,39]. To determine whether CP phosphorylation is extensible to PPV natural hosts, PPV virions were purified from *P. persica* seedlings infected by agroinfiltration with pSN-PPV-5′BD-GFP and analyzed by LC-MS/MS after trypsin/Lys-C digestion and phospho-peptide enrichment.

Fragmentation spectra unambiguously showed phosphorylation in R-CP of *Prunus*-derived virions at three of the four phosphorylatable serines (pS81, pS101, and pS118) and one of the four threonine targets (pT254) previously identified in R-CP produced in herbaceous infections (Figure S2 and Tables S3 and S4). Detection of a phosphorylated version of peptide 1–39 strongly suggested that the fourth serine phosphorylated in *N. bethamiana*, S25, is also modified in peach. However, fragmentation spectra of this peptide did not allow unequivocally ruling out the possibility that, in this case, the phosphate group was modifying residues S16 or T19 instead of S25.

Overall, these results indicated that *O*-GlcNAcylations and phosphorylations affecting PPV virions in vivo were not restricted to experimental herbaceous hosts, once they occurred during infections in the natural host *P. persica*. Moreover, phosphorylation mapping at PPV CP samples from *Prunus* recreated previous results obtained when analyzing virions obtained from experimental herbaceous host species, revealing that this modification largely took place at the same specific residues.

### 3.3. Post-Translational Modifications Affect the CP of Different PPV Strains

Former studies concerning *O*-GlcNAcylation and phosphorylation of PPV CP focused on the isolate PPV-R, belonging to strain D. To assess whether these PTMs affect the CP of other PPV strains, virions from isolates PPV-BOR-3 (strain Rec) and PPV-SwCM (strain C) were analyzed. Rec was selected because signs of phosphorylation and *O*-GlcNAcylation had been previously reported for isolates of this strain [29], and strain C was selected because it is one of the most distant from strain D [43].

PPV-SwCM causes lethal systemic necrosis in *N. benthamiana* [57]. To overcome this constraint, we decided to make use of the construct pICPPV-CPSwCM-R, a chimera that includes the coding sequence of the CP of PPV-SwCM (SwCM-CP) in the backbone of a PPV-R cDNA clone [57], thus allowing expression of SwCM-CP but avoiding deleterious phenotype of this isolate. First, virions purified from *N. benthamiana* plants infected with PPV-BOR-3 and the chimeric plasmid expressing SwCM-CP were subjected to mild trypsin digestion and MALDI-TOF analysis. For SwCM-CP, no tryptic peptides covering the region between amino acids 17 and 72, where presumably *O*-GlcNAcylations would take place, could be recovered. For its part, the poor digestibility of the CP of PPV-BOR-3 (BOR-3-CP) did not allow to obtain useful results using this type of approach either.

**Figure 3:**
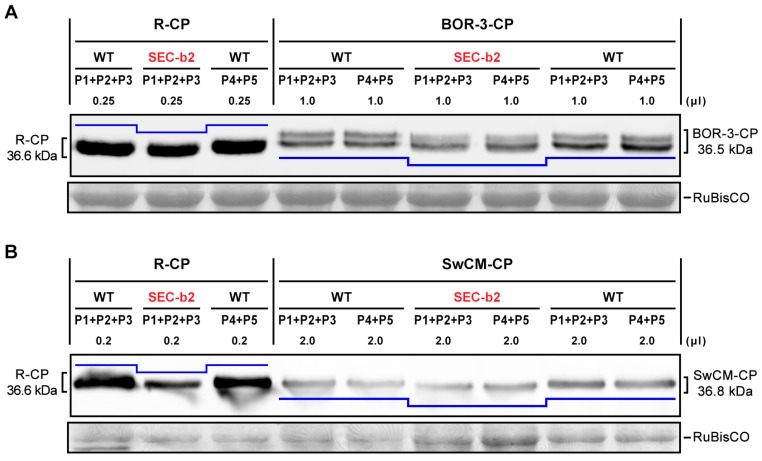
Electrophoretic mobility of the coat protein (CP) of different *Plum pox virus* isolates: PPV-R (R-CP) (**A**,**B**), PPV-BOR-3 (BOR-3-CP) (**A**), and PPV-SwCM (SwCM-CP) (**B**), obtained from *Nicotiana benthamiana* plants, wild-type (WT), and SEC-deficient transgenic plants (SEC-b2). Crude extracts were prepared from systemically infected leaves collected at 17 days post-infection in native conditions and subjected to SDS-PAGE and Western blot anti-CP. Pools of analyzed plants (P1 to P5) and loaded extract volumes are indicated. Blots stained with Ponceau red showing the ribulose-1,5-bisphosphate carboxylase/oxygenase (RuBisCO) large subunit are included as loading controls. Differences in electrophoretic mobility for CPs from WT and SEC-b2 plants are represented with blue lines. This result indicated that the CP of viruses belonging to three different PPV strains was susceptible to be *O*-GlcNAcylated, and this modification was carried out by SEC enzyme.

As an alternative method to qualitatively determine putative *O*-GlcNAcylation of CPs from these isolates, we took advantage of the small differences in electrophoretic mobility in SDS-PAGE previously observed when comparing R-CP produced in wild-type *N. benthamiana* plants with the same protein expressed in *O*-GlcNAcylation-deficient SEC-b2 plants [58]. Such mobility disparity (203 Da per each occupied *O*-GlcNAcylation target) is a consequence of different *O*-GlcNAcylation levels of CP, and, although the difference is subtle, it allows determining whether the OGT SEC is modifying the protein. Extracts from systemically infected leaves of *N. benthamiana* plants, wild type, and SEC-b2, infected with PPV-BOR-3 and PPV-CPSwCM-R, were subjected to SDS-PAGE and Western blot anti-CP. Analysis of CP electrophoretic mobility indicated that—similar to R-CP—BOR-3-CP and SwCM-CP from SEC-b2 plants exhibited greater electrophoretic mobility compared to those from wild-type plants (Figure 3).

As mentioned above, phosphorylation of BOR-3-CP, revealed by its electrophoretic mobility as a doublet in SDS-PAGE, has been previously reported [29]. To confirm BOR-3-CP phosphorylation and map the specific location of modified residues, purified virions of this isolate were analyzed by MS techniques. Two samples of PPV-BOR-3 virions were purified from *N. clevelandii* plants following the method described in Laín et al. [51], and a third sample was purified from *N. benthamiana* plants by a procedure based on that described by Sheveleva et al. [52]. The three samples were digested with trypsin and Lys-C, phospho-enriched, and analyzed by LC-MS/MS.

**Figure 4:**
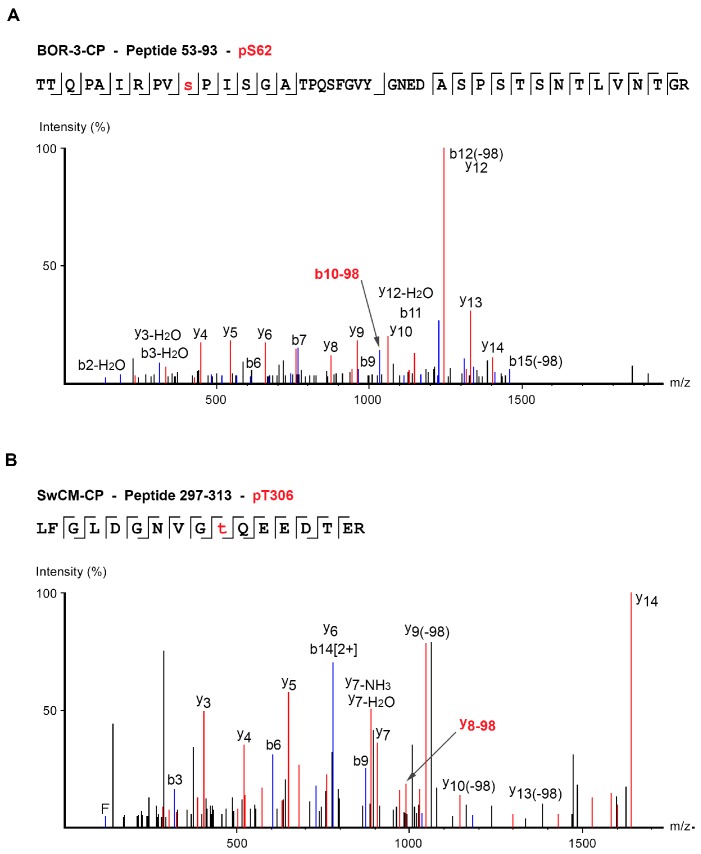
MS/MS fragmentation spectra showing phosphorylations exclusively identified in the coat protein (CP) of two isolates of *Plum pox virus* (PPV): pS62 in PPV-BOR-3 (**A**) and pT306 in PPV-SwCM (**B**).

Collected data allowed mapping phosphorylations at residues S81, S101, S118, and T254 of BOR-3-CP, all of them previously identified in R-CP (Table 1 and Table S4). No evidence linking a particular method of virion purification and success of identification procedure was observed. With the exception of pS118, identified in all three analyses performed, the rest of phospho-targets just could be found in one (pT254) and two (pS81 and pS101) of the three samples, not coinciding. Additionally, another phosphorylation, at serine S62, which had not been found in R-CP, could be mapped. Such identification could be assigned just in one of the processed BOR-3-CP samples when rendering a poor but plausible fragmentation spectra (Figure 4A and Table 1 and Table S4). Although it had not been formally assigned, phosphorylation at S62 had already been predicted by Šubr et al. [59].

**Figure 5:**
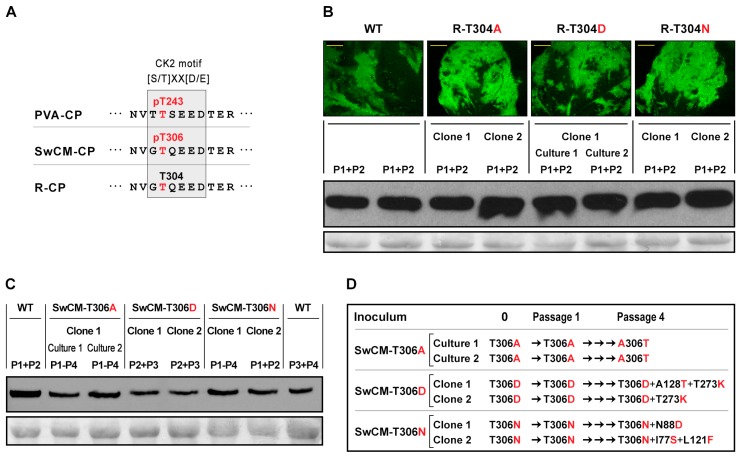
Effect of preventing or emulating phosphorylation at casein kinase 2 (CK2) motif of the coat protein (CP) of *Plum pox virus* (PPV). (**A**) CK2 motif in the CP of *Potato virus A* (PVA-CP), PPV-SwCM (SwCM-CP), and PPV-R (R-CP). The main phosphorylatable residue of PVA-CP (pT243), according to Lõhmus et al. (2017), and the corresponding ones of PPV CP are shown in red. (**B**) Images showing GFP-fluorescence taken under an epifluorescence microscope and CP-specific Western blot analysis of systemically infected leaves of *Nicotiana benthamiana* plants inoculated by hand-rubbing with DNA of pICPPV-NK-lGFP (WT) or the indicated pICPPV-NK-lGFP-derived plasmids, collected at 21 days post-inoculation (dpi). Bars, 5 mm. (**C**) CP-specific Western blot analysis of systemically infected leaves of *Nicotiana benthamiana* plants inoculated by hand-rubbing with DNA of pICPPV-CPSwCM-R (WT) or the indicated pICPPV-CPSwCM-R-derived plasmids, collected at 21 dpi. For both (**B**) and (**C**), inoculations were done with two different clones per construct or, in the case of R-T304D and SwCM-T306A, with two different DNA preparations of a single clone. Pooled plants (P1 to P4) are indicated. Blots stained with Ponceau red showing the ribulose-1,5-bisphosphate carboxylase/oxygenase (RuBisCO) large subunit are included as loading controls. (**D**) Serial passages through *N. benthamiana* plants of viral progenies of PPV-CPSwCM-R-derived mutants. Two lineages were followed for each mutant virus progeny, which were initiated with pools of the two plants inoculated with each DNA sample. Passages were done by hand-rubbing inoculation of two plants using a crude extract of a pool of systemically infected leaves of the two plants inoculated in the former passage, collected at 21 dpi.

To determine whether SwCM-CP is prone to phosphorylation, two replicas of virions purified from *N. benthamiana* plants infected with the chimera PPV-CPSwCM-R were subjected to trypsin/Lys-C digestion and phospho-enrichment and analyzed by LC-MS/MS. Results showed SwCM-CP was phosphorylated at amino acids T83, S120, and T256, respectively, equivalent to phospho-targets S81, S118, and T254 of R-CP and BOR-3-CP (Figure S3) (Table 1 and Table S4, Figure S4). Similar to BOR-3-CP analysis, pS120 was always detected. The other two phospho-targets (pT83 and pT256) were identified in one of the two analyses of SwCM-CP, carried out with the cleaner virion sample. Interestingly, and in contrast to what was observed for the CPs of PPV-R and PPV-BOR-3, residue T306 of SwCM-CP (corresponding to T304 of R-CP and BOR-3-CP) appeared phosphorylated (Figure 4B, Table 1 and Table S4). This residue is located at a CK2 consensus motif initially identified in PVA CP, which is largely conserved among potyviral CPs (Figure 5A, Figure S3, Figure S5, and Figure S6). Although phosphorylation at T306 was detected in only one of the two mapping analysis, it was identified with high confidence by the Peaks search engine.

Taken together, these results showed that CP phosphorylation was widespread among PPV strains and affected protein sites in either ubiquitous or specific way.

### 3.4. The Relevance of Phosphorylation at Conserved CK2 Motif of CP Differs among Potyviruses

Phosphorylation at the CK2 motif of CP has been shown to take place during PVA infection, and mutations that either prevent or emulate phosphorylation at T243 in this motif are deleterious for viral infection [24,26].

The detection in PPV of phosphorylated T306 at the CK2 motif of SwCM-CP suggested that phosphorylation at CP CK2 motif could be relevant for PPV infection, even though the equivalent residue in R-CP (T304) (Figure 5A) had never been found modified.

To explore this possibility, mutations were introduced in pICPPV-NK-lGFP and pICPPV-CPSwCM-R, to replace residues T304 of R-CP and T306 of SwCM-CP by alanine (R-T304A, SwCM-T306A) or aspartic acid (R-T304D, SwCM-T306D) to, respectively, prevent or emulate phosphorylation at those sites. Aspartic acid serves to mimic steric and electrostatic effects of phosphorylation without suffering such modification. Replacements by asparagine, a neutral amino acid with a bulky side chain similar to the negatively charged aspartic, were included as controls (R-T304N, SwCM-T306N) (Figure S1).

In a first experiment, DNA of R-T304A, R-T304D, R-T304N, or non-mutated pICPPV-NK-lGFP was mechanically inoculated in *N. benthamiana* plants by hand-rubbing, and symptoms of infection and GFP fluorescence expressed from recombinant viruses were monitored. Tracking viral infection for up to 21 dpi did not show differences in infectivity or progression of infection between the different mutants and the wild-type virus (Table S5). Furthermore, at that time, no major differences in GFP fluorescence or viral accumulation were detected in systematically infected leaves, as assessed by Western blot anti-CP (Figure 5B). Amplification by IC-RT-PCR followed by sequencing of a cDNA fragment corresponding to the entire CP sequence confirmed the stability of the three mutations in each of the viral progenies. Following this, with the objective of facilitating the putative adaptation of mutants R-T304A, R-T304D, and R-T304N, the progeny of each mutant was subjected to four serial passages through *N. benthamiana* plants. After the fourth passage, analysis of viral progenies confirmed the stability of all mutations and absence in the CP coding sequence of mutations distinct to those originally engineered. These results indicated that—contrary to what happens in PVA CP—preventing or emulating phosphorylation at CK2 motif of R-CP did not appear to significantly affect viral infection, and suggested that phosphorylation at this motif was not relevant for all potyviruses.

Next, we analyzed the effect of disturbing phosphorylation at the CK2 motif of SwCM-CP. DNAs of SwCM-T304A, SwCM-T304D, and SwCM-T304N were inoculated into *N. benthamiana* plants, using as control the non-mutated pICPPV-CPSwCM-R chimera. Most plants inoculated with the wild-type virus (8/8) or with mutant SwCM-T306A (7/8) showed signs of infection at an early time (12 dpi); while the number of plants showing symptoms after being inoculated with mutants SwCM-T306D or SwCM-T306N (1/8 and 0/8, respectively) was clearly smaller at that time. However, after 21 dpi, infection symptoms of all mutants tended to equalize (Table S5). At that time, viral accumulation in systemically infected tissue was found to be slightly lower in plants inoculated with SwCM-T306D than in those infected with any of the other two mutants or the wild-type virus (Figure 5C). IC-RT-PCR and later sequencing of the CP coding region of viral populations in plants infected with each construct showed no sequence alterations in any case.

Slowing-down in the progression of infection observed after inoculating with mutants SwCM-T306N and SwCM-T306D, along with the lower viral accumulation detected for this last one, suggested that modification of phospho-target T306 could affect the biological efficacy of the pathogen. As before, to facilitate a putative evolutionary adaptation of SwCM-T306 mutants, which could provide information about their potential defects, the progeny of each mutant was subjected to serial passages. After four passages, analysis by IC-RT-PCR and sequencing of the CP coding region revealed the emergence of spontaneous mutations in viral progenies of all mutants (Figure 5D). In two lineages of the SwCM-T306A mutant, a single nucleotide change (**G**CA X **A**CA) allowed a complete reversion of alanine to threonine at position 306, emulating wild-type SwCM-CP sequence. In turn, the two analyzed lineages of the SwCM-T306D mutant showed a mutation (ACA X A**A**A), causing a change from threonine to lysine at position 273. In one of these lineages, a second mutation (GCC X **A**CC) appeared, which resulted in a change from alanine to threonine at position 128. Finally, each of SwCM-T306N lineages took a different evolutionary path. In one of them, a point mutation (AAT X **G**AT) was introduced, causing a change from asparagine to aspartic acid at position 88; while in the other, mutations ATT X A**G**T and CTC X **T**TC, respectively, produced changes from isoleucine to serine at position 77 and from leucine to phenylalanine at position 121 (Figure 5D).

Taken together, these results suggested that modifying the phospho-target at CK2 motif of SwCM-CP did indeed cause a deleterious effect on viral infection in the case of PPV strain C. However, such an effect was not as drastic as that previously reported for mutations at CK2 motif of PVA-CP, and it did not extend to PPV isolates of strain D.

## 4. Discussion

The function of the coat protein of potyviruses, which is a multitasking protein essential for viral infection, goes beyond forming stable virion structures to protect genomic RNA [19]. For instance, the CP of the potyvirus *Tobacco etch virus* (TEV) has been shown to be essential for efficient viral movement, but functions of TEV CP in virion assembly and movement appeared to be somewhat distinct [60]. Moreover, potyviral CP has been suggested to have regulatory roles by adjusting viral RNA allocation and availability to participate at different steps of viral infection [24,30], particularly in translation/replication transition [26]. The suppression of the pathogen-associated molecular pattern-triggered immunity is another contribution to the viral infection described for the CP of a potyvirus [61]. On the other hand, the potyviral CP is itself target of host defense responses, and escaping them by evolving CP sequence may condition the viral host range [62,63,64,65]. PTMs not only expand proteome size but also provide individual proteins with greater functional dynamism. This especially applies to phosphorylation and *O*-GlcNAcylation, two very frequent and closely related PTMs [31,66]. Thus, it is not unexpected that these PTMs have been found in a protein, such as potyviral CP, responsible for coordinating so many important functions [25,29,67]. However, such modifications do not seem to affect all potyviruses, and their effect may be different for each viral species. Indeed, the CP of PPV displays both *O*-GlcNAcylations and phosphorylations, while that of PVA is only phosphorylated. Moreover, PPV and PVA CP phosphorylations have shown specific peculiarities [26,38,39]. In this work, we deepened into the characterization of PTMs affecting PPV CP, showing they are not exclusive to the protein assembled in virions and occur not only in experimental herbaceous plants but also in natural woody PPV hosts. We also revealed common and specific features of phosphorylation modifying CP of different PPV isolates.

The widely studied phosphorylation of PVA CP has not been reported to occur in virions [25]. This contrasts with phosphorylations and *O*-GlcNAcylations of PPV, which have been exclusively studied on purified virus particles [37,39]. In this work, we demonstrated that these two modifications also affected PPV CP not assembled in mature virus particles (Figure 1). Whereas, these results suggested that PTMs of PPV CP preceded virion formation; they did not exclude the possibility that they might also directly affect CP already assembled in virions. Importantly, although both phosphorylation and *O*-GlcNAcylation are reversible PTMs, enzymatic removal of phosphate or *O*-GlcNAc groups from potyviral CPs has not been demonstrated. If these reverse reactions indeed take place, we do not know if they occur on free or assembled CP, or at both states of protein aggregation. The MALDI-TOF approach used here did not allow identification of which specific residues are modified by *O*-GlcNAcylation. However, analysis of peptide 1–39 seemed to indicate that the same amino acids previously mapped in CP of purified virions (T19 and T24) were modified in not-fully assembled CP. This would suggest that the *O*-GlcNAcylation of PPV CP does not depend on the assembly level of the protein. The scope of our phosphorylation analysis is also limited. It clearly demonstrates this PTM can take place in CP not assembled in mature virions, but it only provides information about a PPV-BOR-3-specific phosphorylation target (see below). This is especially important because very different functions have been suggested for phosphorylations at different locations of PPV CP [39]. To be able to draw definitive conclusions about relationships between phosphorylation and the assembly state of PPV CP, it will be necessary to have a precise phospho-mapping in CP that is not forming particles.

PTMs of viral proteins usually involve host enzymes and, thus, they could depend on host-specific peculiarities. Phosphorylation and *O*-GlcNAcylation of PPV CP have been previously studied in herbaceous experimental hosts exclusively; thus, analyzing them in *P. persica* has the interest of extending our knowledge to a quite different plant species and bringing it closer to natural conditions. Phosphorylation mapping in PPV virions from *P. persica* confirmed this modification also comes about in this host (Figure S2 and Table S3). Although poor sequence coverage (around 50%) impeded confirming the status of all phosphorylatable targets previously identified in *Nicotiana* spp., this modification was detected in most of them, including major targets in the protein core with significant influence in viral infection, pS118 and pT254. Phosphorylation was also detected in targets of the N terminus of the protein, pS81, pS101, and, probably, pS25. This result suggested that both functions attributed to PPV CP phosphorylation were conserved in herbaceous and woody hosts: the fine-tuning of protein stability by phosphorylation in coordination with *O*-GlcNAcylation at N-terminal disordered protrusion of the protein, as well as the control of virion stability carried out by phosphorylation at the core region of the protein [39].

Here, we demonstrated that PPV CP *O*-GlcNAcylation also took place in both herbaceous plants and in the natural PPV host *P. persica* (Figure 2). MALDI-TOF patterns of peptides 1–39, 40–93, and 30–108 (Figure 2A) seemed to be similar to those previously reported for PPV CP produced in *Nicotiana* spp. [37,68]. This suggested that the amino acids targeted by *O*-GlcNAcylation could be the same for both hosts. The possible higher *O*-GlcNAcylation level of CP in *P. persica* PPV infections (Figure 2B) could be consistent with the reduced fitness in *P. persica* of PPV NAT, a natural deletion mutant that lacks two *O*-GlcNAcylation targets (gT19 and gT24) [46]. However, this would contrast with the fact that replacement of all *O*-GlcNAcylable threonines of PPV CP by alanines has comparable effects on infections of *P. persica* and herbaceous hosts [37]. Therefore, although *O*-GlcNAcylation of CP appears to be important for PPV in both experimental herbaceous plants and natural woody hosts, it is not essential for infection in any of these plants.

PPV is a virus with high intra-species variability. At least nine PPV strains have been identified so far [43,69], with the largest divergence affecting the coding sequence of the N-terminal region of CP, where all *O*-GlcNAcylation and most of the phosphorylation targets are focused. Thus, we wondered if PTMs widely analyzed in an isolate (PPV-R) of the strain D would similarly affect other PPV strains. Sequence alignment of CPs from isolates of different PPV strains showed that most potential targets of *O*-GlcNAcylation in PPV-R virions (T19, T24, T41, T50, T53, and T54) were conserved in the other two isolates analyzed in this study, PPV-BOR-3 (strain Rec) and PPV-SwCM (strain C). While S65 was not conserved in SwCM-CP, T58 was not conserved in either SwCM-CP or BOR-3-CP (Figure S3).

Poor protease digestibility and tryptic peptide coverage of the N-terminal region of BOR-3- and SwCM-CP precluded obtaining sound results from MALDI-TOF analyses. However, qualitative analyses exploiting variations in electrophoretic mobility of differentially modified protein allowed confirming that CP *O*-GlcNAcylation by SEC occurred in viral strains different to D (Figure 3). Nevertheless, some variability in *O*-GlcNAcylatable targets could not be ruled out.

Furthermore, almost all phosphorylatable serine/threonine identified in the CP of PPV-R (T71, S81, S101, S118, T254, and T313) were conserved in PPV-BOR-3 and PPV-SwCM, while phospho-targets aligning with S25 and S106 were absent from the isolate of strain Rec (Figure S3). Some phosphorylations equivalent to those previously detected in R-CP were found in the N-terminal protrusion and the core of the BOR-3-CP (pS81 and pS101, and pS118 and pT254, respectively) and SwCM-CP (pT83, and pS120 and pT256, respectively) (Table S4). This suggested that different functions attributed to phosphorylation in each CP region could be largely conserved among PPV viral strains.

Our LC-MS/MS analyses showed phosphorylations in the CP of PPV-BOR-3 and PPV-SwCM modifying residues not equivalent to those previously identified as phospho-targets in PPV-R CP (Table S4). Phosphorylation of S62 at BOR-3-CP (Figure 4) was previously guessed by Šubr et al. [59] as presumed responsible for the typical, and exclusive, double-band phenotype, characterizing the CP of this isolate in SDS-PAGE assays. Awaiting some experimental evidence, we could provisionally conjecture that phosphorylation in S62 could contribute to the coordinated phosphorylation/*O*-GlcNAcylation role in protein stability suggested for the PTMs of the N-terminal protrusion of PPV CP, based on former results obtained with PPV R [39].

Moreover, we detected the phosphorylated residue T306 in SwCM-CP (Figure 4). This amino acid is equivalent to T304 of R-CP, which has never been found phosphorylated, and to T243 of PVA, placed in the CK2 motif (Figure 5A) whose phosphorylation has been shown to be essential for PVA replication [26]. Absence of phosphorylation in T304 PPV-R CP agreed with the observation that mutations that prevent or mimic phosphorylation at this residue did not show noticeable deleterious effects and were stable after several serial passages (Figure 5B). In contrast, although phosphorylation-related mutations affecting T306 of SwCM-CP did not cause severe defects in initial viral infections, they either reverted or prompted the emergence of allegedly compensatory second mutations, thus evidencing that altering T306 phosphorylation has a significant fitness cost. It seemed, therefore, that phosphorylation at the CK2 motif could have different impacts on the viral cycle even between strains of the same potyvirus. Effects of disturbing phosphorylation in T306 of SwCM-CP were reminiscent of those described for the CK2 motif of PVA-CP; in PVA, however, both types of mutations, preventing or mimicking phosphorylation at this motif, were much more damaging, causing a complete block of viral replication [26]. In any case, phosphorylation at the CP CK2 motif appeared to be less relevant for PPV than for PVA. In the case of PPV, the role of CK2 motif phosphorylation could be compensated by other phosphorylations or structural changes at different CP locations.

It has been suggested that phosphorylation at CP residue T243 of PVA-CP by protein kinase CK2 plays a crucial role in RNA translation/replication transition. Phosphorylation of T306 at the CK2 motif-like of SwCM-CP could have a similar function, but in this case, the contribution would be much less significant. However, we could also envisage the possibility that phosphorylation of T306 is involved in a function related to that proposed for phosphorylation at the core domain of R-CP in the control of virion assembly/disassembly. Moreover, we could not rule out another possible role, still non-characterized, specific for the CP of this particular PPV strain.

Outcomes described in this paper, along with previously reported data, demonstrate that PTMs are general features of particular potyviruses, whose relevance varies between different members of this virus group. The fact that neither phosphorylation nor *O*-GlcNAcylation appears to be essential for PPV infection agrees with a recent acquisition of these PTMs in potyvirus evolution. The divergences on features and impact of phosphorylation of CPs of PPV and PVA CPs also support this assumption. On the other hand, conservation of these PTMs among different PPV strains suggests that they were acquired soon in PPV evolution, undergoing strain-specific adjustments throughout the diversification of the species. The preservation of phosphorylation and *O*-GlcNAcylation during the evolutionary history of PPV provides clear evidence of its usefulness for the virus. We presume that PTMs provide the chance to carry out varied improvements on the regulatory capacity of multifunctional proteins as potyviral CP, in a manner that they can be specifically adopted by different viruses at different evolutionary stages.

## Figures and Tables

**Table 1:** Phosphorylation mapping in the coat protein (CP) of isolates BOR-3 and SwCM, after processing virions of *Plum pox virus* by LC-MS/MS.

Viral Isolate	Peptide Sequence	Start-End	Modification Site	z	*m*/*z*	Search Engine
**BOR-3**	TTQPAIRPVpSPISGATPQSFGVYGNEDASPSTSNTLVNTGR	53–93	**S62**	4	1064.51	Peaks, score > 50
	SFGVYGNEDApSPSTSNTLVNTGR	71–93	**S81**	3	818.35	Mascot, score > 50 Peaks, score > 50
	GpSIGTFAVPR	100–109	**S101**	2	542.76	Mascot, score > 50 Peaks, score > 50
	TMTSKLpSLPK	112–121	**S118**	2	593.30	Mascot, score > 40 Peaks, score > 50
	NLpTDYSLAR	252–260	**T254**	2	566.75	Mascot, score > 30 Peaks, score > 50
**SwCM**	QNVpTPSSSNALVNTR	80–94	**T83**	2	834.39	Mascot, score > 50 Peaks, score > 50
	SMTSKLpSLPK	114–123	**S120**	2	586.29	Mascot, score > 25 Peaks, score > 50
	NLpTDYSLAR	254–262	**T256**	2	566.75	Mascot, score > 35 Peaks, score > 50
	LFGLDGNVGpTQEEDTER	297–313	**T306**	3	653.95	Mascot, score > 30 Peaks, score > 50

MS/MS fragmentation spectra of listed phospho-peptides are shown in Figure 4 and Figure S3.

## Supplementary Materials

The following are available online at https://www.mdpi.com/1999-4915/12/3/308/s1.
