# Supplementary material for: Common and Strain-Specific Post-Translational Modifications of the Potyvirus Plum pox virus Coat Protein in Different Hosts
Source: Viruses. 2020 Mar 12;12(3):308. doi: 10.3390/v12030308 (PMC7150786; doi:10.3390/v12030308)
Supplement: Supplementary file 1 [file viruses-12-00308-s001.pdf]

SUPPLEMENTARY INFORMATION

SUPPLEMENTARY FIGURES

Figure S1

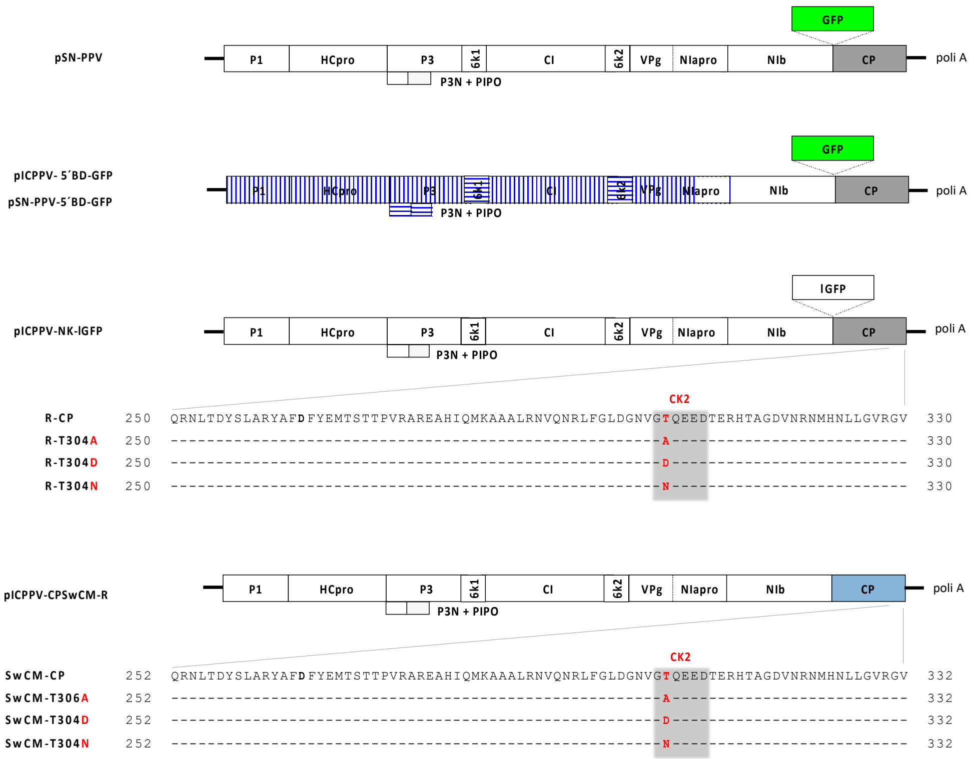

Schematic representation of PPV full-length cDNA clones. Sequences of C-terminal region of PPV-R and PPV-SwCM CPs are displayed below corresponding constructs. Mutations engineered into the casein kinase II (CK2) motif (yellow background) in constructs pICPPV-NK-lGFP and pICPPV-CPSwCM-R are shown in red.

Figure S2

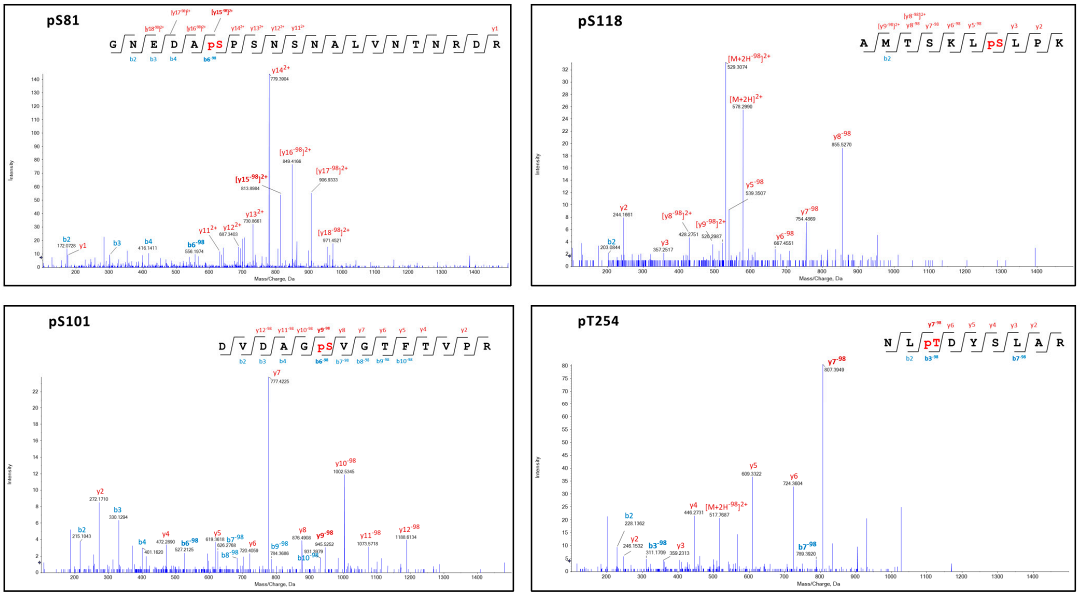

MS/MS fragmentation spectra identifying phosphorylations at S81, S101, S118 and T254 in the capsid protein of PPV-R virions purified from *Prunus persica* plants. Details of identified phospho-peptides are provided in Supplementary Table S3.

Figure S3

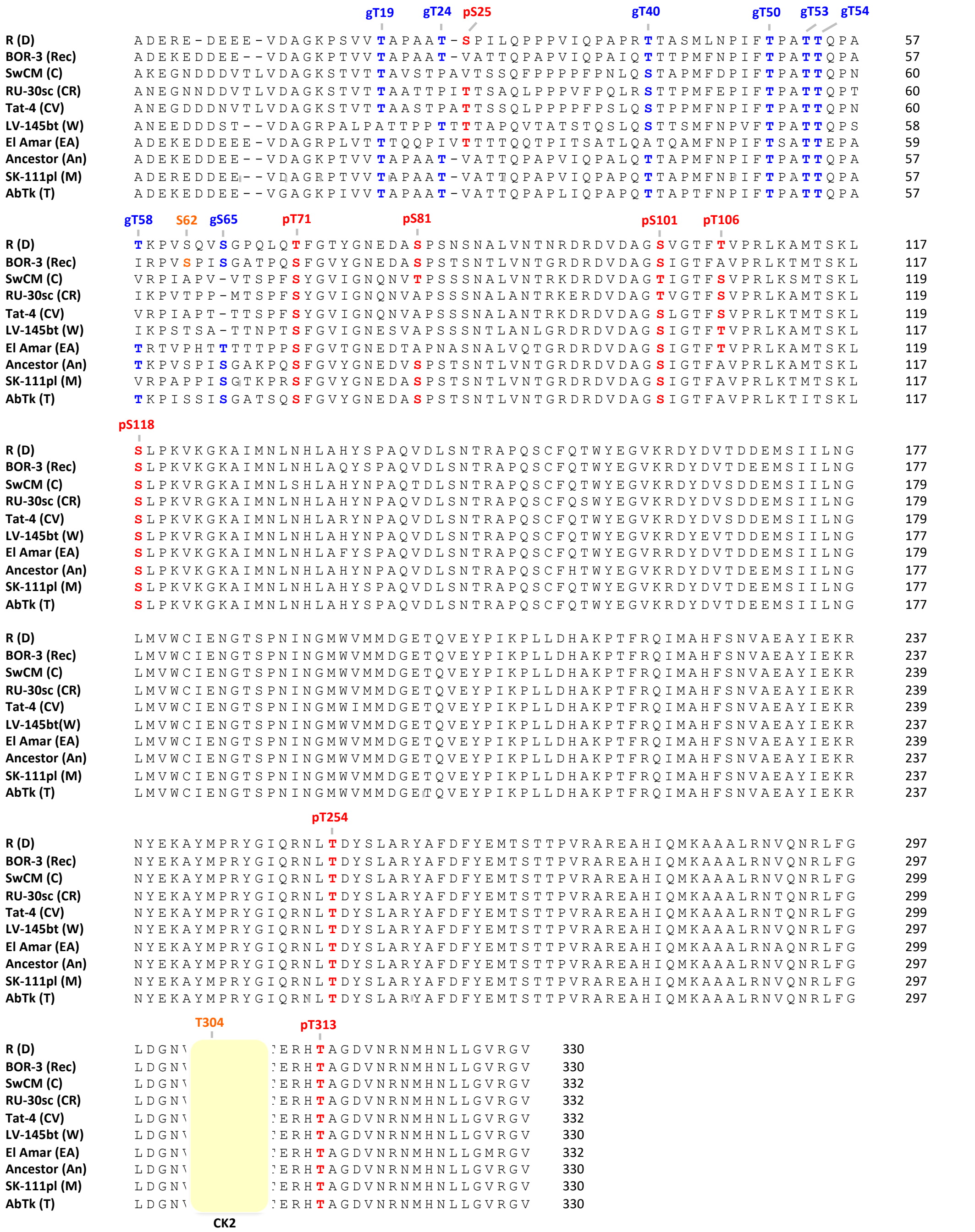

Supplementary Figure S3: Alignment of coat protein (CP) sequences from representative isolates of the ten strains proposed for *Plum pox virus* (PPV). Respective strains are in parentheses. Accession numbers for the aligned sequences are: R, EF569215; BOR-3, AY028309; SwCM, sequence not deposited in a public repository; RU-30sc, KC020126; Tat-4, MF447180; LV-145bt, HQ670748; El Amar, AM157175; Ancestor, HF674399; SK-111pl, HF585099; AbTk, EU734794. Residues in which phosphorylations (red) and *O*-

GlcNAcylations (blue) had been previously identified in the CP of PPV-R virions [1-4], as well as the equivalent residues, potentially modifiable in other isolates, are highlighted. Amino acids found phosphorylated just in the CP of BOR-3 (pS62) and SwCM (pT306) are highlighted in orange. Casein kinase II (CK2) motif, largely conserved among potyviruses, is shown on a yellow background. Proteins were aligned using Clustal Omega program (*European Bioinformatics Institute*).

Figure S4

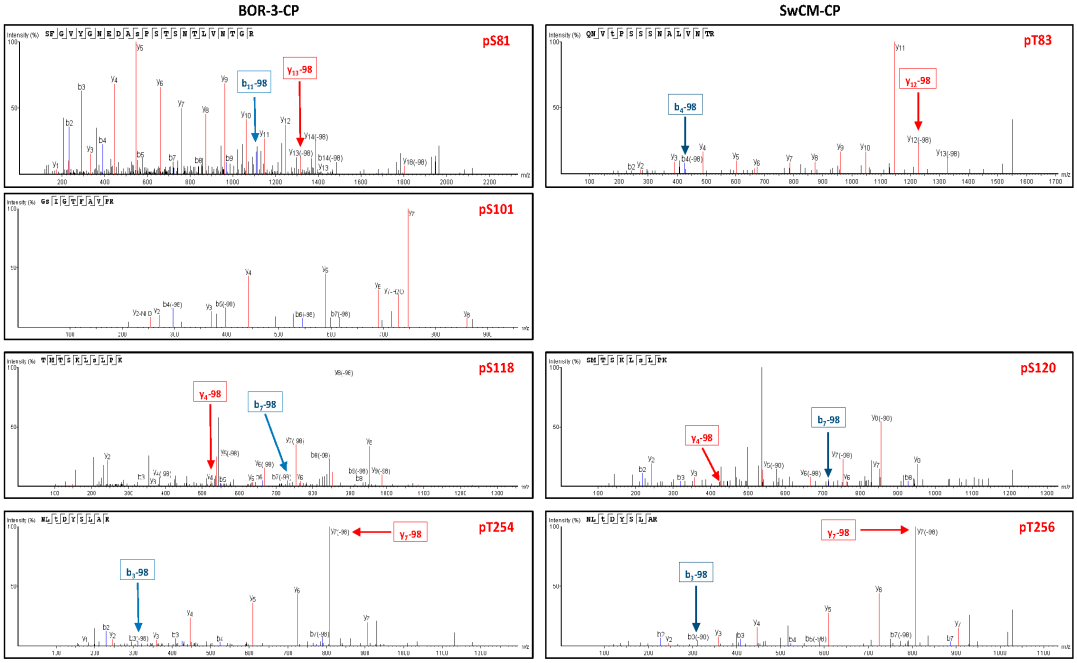

MS/MS fragmentation spectra showing phosphorylations in the coat protein (CP) of PPV-BOR-3 (BOR-3-CP) and PPV-SwCM (SwCM-CP) virions. Details of phospho-peptides corresponding with these spectra are provided in Table 1.

Figure S5

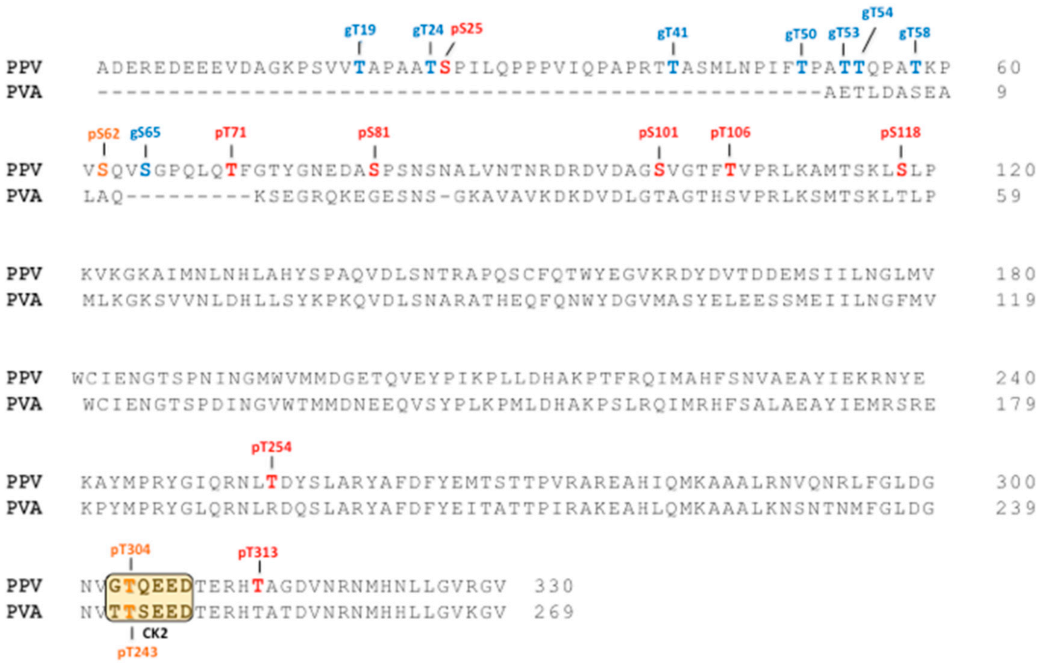

Alignment of coat protein (CP) sequences from the *Plum pox virus* (PPV) isolate PPV-R (EF569215) and *Potato virus A* (CAC17411). Residues in which phosphorylations (red) and *O*-GlcNAcylations (blue) had been identified in the CP of PPV-R virions [1-4] are highlighted. Amino acids equivalents to those residues found phosphorylated in the CPs of PPV-BOR3 and PPV-SwCM (see Figure S3) are highlighted in orange. Casein kinase II (CK2) motif, largely conserved among potyviruses, is shown on a yellow background. Proteins were aligned using Clustal Omega program (*European Bioinformatics Institute*).

Figure S6

Virus name	Acronym	Id	282	CK2 motif	329
Plum pox virus (isolate R)	**PPV**	EF569215	QMKAAALRNVQNRLFGLDGNV	**GTQEED**	TERHTAGDVNRNMHNLLGVRG
Algerian watermelon mosaic virus	**AWNV**	YP_001931956.1	QMKAAAIRGGNRRLFGIDGNV	**GGGDEN**	TERHTVDDVERDMHSLLGMRK
Banana bract mosaic virus	**BBrMV-B**	YP_001427389.1	QMKAAAIRGSNTRLFGLDGNL	**GPGEEN**	TERHTVEDVKRDMHSLLGMKH
Bean common mosaic necrosis virus	**BCMNV**	BBH10738.1	QMKAAALANVNTRLFGLDGNV	**ATTSEN**	TERHTARDVNQNMHHLLGMTS
Bean common mosaic virus	**BCMV**	AAB28498.1	QMKAARLANVNTRLFGLDGNV	**ATTSEN**	TERHTARDVNQNMHHLLGMTS
Bean yellow mosaic virus	**BYMV-J**	NP_612218.1	QMKAAAVRGKSTRLFGLDGNV	**GTDEEN**	TERHTAGDVNRDMHTMLGVRI
Beet mosaic virus	**BtMV**	AAF80972.1	QMKAAALASVSNKLFGLDGSA	**DTTSED**	TERHTATDVNAHMHHMMGVRQ
Bidens mosaic virus	**BiMV**	AAX63416.1	QMKAAALKSSQTRMFGLDGGI	**GTQTEN**	TERHTTEDVSPNMHTLLGVRN
Blackberry virus Y	**BVY**	YP_851006.1	QMKAAALESASSKVLGLDGGS	**A-RAID**	TERHTTEDATARTHNLRGAAM
Canna yellow streak virus	**CaYSV**	AIX02891.1	QMKAASIRGGTNNLFGLDGNV	**GEDSEN**	TERHVATDVNKNTHSYRGAQI
Celery Mosaic Virus	**CeMV**	YP_004376199.1	QMKAAALRNTKTRLLGLDGSV	**SGKEED**	TERHTVDDVNRNMHSLLGMQG
Chilli ringspot virus	**ChiRSV**	YP_004875339.1	QMRAAAVMHAKNNMFGLDGNV	**STKEEN**	TERHTATDVNRDMHHLMGVRG
Clover yellow mosaic virus	**ClYMV-1**	NP_613273.1	QMKAAAIRGKSNHMFGLDGNV	**GTDEEN**	TERHTANDVNRNMHHIAGARF
Cucurbit vein banding virus	**CVBV**	YP_009388623.1	QMKAAALSNVKTRMFGLDGSV	**GNSEEN**	TERHTAHDVNQNLHTLMGVRG
Daphne mosaic virus	**DapMV**	YP_610949.1	QMKAAALRNASTHLFGLDGNV	**GNAAEN**	TERHTTDDVDVNTHSFTGARM
Daphne virus Y	**DVY**	YP_009508399.1	QMKAAALKNASTRMFGLDGKV	**GTATEN**	TERHTADDVNANTHSLSGARI
Dasheen mosaic virus	**DsMV**	QBI77037.1	QMKAAALSNVTTRLFGLDGNV	**STSSEN**	TERHTAKDVTPNMHTLLGVSP
Dendrobium chlorotic mosaic virus	**DeCMV**	QBS16347.1	QMKAAALVNVSTKLFGLDGNI	**GTQEED**	TERHVAIDVNRNMHTLLGMRH
Dioscorea mosaic virus Florida	**DMV-FL**	AZB50213.1	QMKAGALRGVTTKLFGIDGKI	**RNSEED**	TEHPTSGDVSRNMPHFLGERG
East asian passiflora virus	**EAPV**	BAR88501.1	QMKAAALANVSTRLFGLDGNV	**STTGEN**	TERHTARDVNQNMHTLLGMNS
Euphorbia ringspot virus	**EuRSV**	YP_009305422.1	QMKAAALRNVKNNLFGLDGNV	**GSKEEN**	TERHTADDVNRNMHSLLGMQA
Freesia mosaic virus	**FreMV**	YP_003587807.1	QMKAAAVANTTAKMFGLDGNI	**GTQEEN**	TERHTVTDVSANMHSLLGVRH
Gomphocaarpus mosaic virus	**GoMV**	BBD33993.1	QMKAAALVNTNTRLFGLDGNV	**TTSEEN**	TERHVATDVNANMHTLLGVRQ
Habenaria mosaic virus	**HaMV**	YP_008240477.1	QMKAAALRNTSSRMFGLDGKV	**GTQVED**	TERHTAEDVNRNMHNLLGVRG
Hubei poty-like virus 1	**HuPLV1**	APG79040.1	QMKAAAVRGKSNRLFGLDGNV	**GTDEEN**	TERHTAGDVNRNMHTMLGVRI
Japanese yam mosaic virus	**JYMV**	AJD23401.1	QMKAAALRGVQNKLFGLDGNV	**STMEEN**	TERHTAEDVNRNMHSLLGVRG
Johnsongrass mosaic virus	**JGMV**	ALS88434.1	QMKAAAIRGSTNHMFGLDGNV	**GESSEN**	TERHTAADVSRNVHSYRGAKI
Keunjorong mosaic virus	**KjMV**	YP_004934107.1	QMKAAALVNTNTRLFGLDGNV	**STIEEN**	TERHVATDVNANMHTLLGVRQ
Konjac mosaic virus	**KoMV**	YP_529485.1	QMKASALRSANTRMFGLDGKV	**TTKEED**	TERHTAEDVTRNLHTLMGVRA
Lettuce italian necrotic virus	**LINV**	YP_009162372.1	QMKAAALRNASTRLFGLDGNV	**GTKEEN**	TERHTAEDVNSTMHHLLGMRG
Lettuce mosaic virus	**LMV**	NP_619667.1	QMKAAALVGTQNRLFGMDGGG	**STQEEN**	TERHTAADVNQNMHTLLGVRG
Lily yellow mosaic virus	**LYMV**	YP_009553508.1	QMKAAALRNARNTLFGLDGNV	**GTAEED**	TERHTAQDVNRNMHNLLGVRM
Maize dwarf mosaic virus	**MDMV**	NP_569138.1	QMKAAAVRGSNTRMFGLDGNV	**GETQEN**	TERHTAGDVSRNIHSLLGVQQ
Moroccan watermelon mosaic virus	**MWMV**	YP_001552410.1	QMKAAAIRGANTRLFGIDGNV	**GGGEEN**	TERHTVDDVERDMHSLLGMRK
Onion yellow dwarf virus	**OYDV**	BAE96765.1	QMKAAAIRGATNRLFGLDGNV	**NTTEED**	TERHTAADINKNQHTLLGIKM
Panax virus Y	**PanVY**	YP_003725718.1	QMKAAALKNSRTRLFGLDGSV	**SSKEED**	TERHTVEDVNRNMHSLLGMQG
Papaya leaf distortion mosaic virus	**PLDMV**	NP_870995.1	QMKAAALRDANNKMFGLDGKV	**GNATEN**	TERHTADDVNHNTHAFTGVRY
Papaya ringspot virus	**PRV**	AAB23789.1	QMKAAALRNTSRRMFGMDGSV	**SNKEEN**	TERHTVEDVNRDMHSLLGMRN
Paris mosaic necrosis virus	**PMNV**	AUB51246.1	QMKAAALTSVNNKLFGLDGNV	**STTSEN**	TERHTARDVNQNMHSLLGMTS
Passionfruit Vietnam virus	**PVNV-DakNong**	AZF86246.1	QMKAAALTGTVNKLFGLDGNV	**GTTSED**	TERHTARDVNQNMHTLLGVGP
Pea enation mosaic virus	**PeMV**	AAC42975.1	QMKAAAVRGSNTRMFGLDGNV	**GESQEN**	TERHTAGDVSRNMHSLLGVQQ
Pea seed-borne mosaic potyvirus	**PSbMV**	QBL54815.1	QMKAAAIRGKSNSLFGLDGNV	**GTQEEN**	TERHTAEDVNQNMHNLLGMRA
Peanut mottle virus	**PEMVM-1**	NP_068348.2	QMKAAALNNVAIKTFGLDGNV	**GTQDED**	TERHTANDVNRNMHSLLGMRQ
Peanut stripe virus	**PStV**	AGZ92019.1	QMKAAALSNVNSKLFGLDGNV	**ATTSEN**	TERHTARDVNQNMHTLLGMGS
Pecan mosaic-associated virus	**PMaV**	YP_009256204.1	QMKAAALRGSSNRMFGLDGKA	**NTEGEN**	TERHTTDDVKQDMHSLLGVIN
Pennisetum mosaic virus	**PeMV**	YP_249455.1	QMKAAAVRGSNTRMFGLDGNV	**GESQEN**	TERHTAGDVSRNMHSLLGVQQ
Pepper mottle virus	**PepMOV**	NP_041276.1	QMKAAALKSAQTRLFGLDGGI	**GTQGEN**	TERHTTEDVSPDMHTLLGVRE
Pepper severe mosaic virus	**PepSMV**	YP_778468.1	QMKAAALKSVQTRMFGLDGGI	**STQEEN**	TERHTTEDVSPHMHTLLGVRN
Pepper yellow mosaic virus	**PepYMV**	YP_003778216.1	QMKAAALKSAQTRMFGLDGGI	**STQEEN**	TERHTTEDVSPNMHTLLGVRN
Platycodon mild mottle virus	**PlaMMV**	AYA60486.1	QMKAAAIRNAHNRLFGLDGNV	**GTTEED**	TERHTANDVRRNVHHLMGAQL
Pleione flower breaking virus	**PlFBV**	YP_009552772.1	QMKAAALNNVSTRLFGLDGNV	**VTQEEN**	TERHTTADVNANMHSLLGVRH
Pokeweed mosaic virus	**PkMV**	YP_008719787.1	QMKAAALRNASTKMFGLDGNV	**GTKEEN**	TERHTAEDVSREMHNLMGVRM
Potato virus A	**PVA**	CAC17411.1	QMKAAALKNSNTNMFGLDGNV	**TTSEED**	TERHTATDVNRNMHHLLGVKG
Potato virus Y	**PVY**	BAN16607.1	QMKAAALKSAQSRLFGLDGGI	**STQEEN**	TERHTTEDVSPSMHTLLGVKN
Potato yellow blotch virus	**PYBV**	AFS28882.1	QMKAAALKNANTSMFGLDGNV	**TTSEED**	TERHTASDVNRNMHHLLGVKG
Saffron latent virus	**SalV**	YP_009455737.1	QMKAAALTNVDSRLFGLDGNA	**STNLEN**	TERHTAKDVNQNMHTLLGMGS
Shallot yellow stripe virus	**SYSV**	YP_331412.1	QMKAAAVRGVANRMFGLDGNI	**STDDEN**	TERHTAADVNKDHHTLFGLRM
Soybean mosaic virus	**SMV**	AAB22819.2	QMKAAALSGVNNKLFGLDGNI	**STNSEN**	TERHTARDVNQNMHTLLGMGP
Sudan watermelon mosaic virus	**SuWMV**	YP_009407951.1	QMKAAAIRNTNRRMFGIDGSI	**GGGEEN**	TERHTVDDVERDMHSLLGMRK
Sugarcane mosaic virus	**SCMV-Sp**	CAJ33884.1	QMKAAAVRGSNTRLFGLDGNV	**GETQEN**	TERHTAGDVSRNMHSLLGVQQ
Sunflower chlorotic mottle virus	**SuCMoV**	ADF31932.1	QMKAAALKSAQTRMFGLDGGI	**GTKEEN**	TERHTTEDVNPNMHTLLGVRN
Sunflower ring blotch virus	**SuRBV**	YP_009351870.1	QMKAAALKSAQTRMFGLDGGI	**STQEEN**	TERHTTEDVSPNMHTLLGVRN
Sweet potato feathery mottle virus	**SPFMV**	BAJ04334.1	QMKAAALKNARNRLFGLDGNV	**STQEED**	TERHTTTDVTRNIHNLLGMRG
Sweet potato latent virus	**SPLV**	YP_007697620.1	QMKAAALTNTHHRLFGLDGNV	**STTEEN**	TERHTATDVDRNIHTLLGMRG
Sweet potato virus 2	**SPV2**	YP_006382460.1	QMKAAALKNAQNRLFGLDGNI	**STQEED**	TERHTTTDVTRNIHNLLGMRG
Sweet potato virus G	**SPVG**	AFJ68039.1	QMKAAALKNAQNRLFGLDGNV	**STQEED**	TERHTTTDVTRNIHNLLGMRG
Telosma mosaic virus	**TeMV**	YP_001427386.1	QMKAAALVGTTNRMFGSDGSV	**STACED**	TERHTARDVNQNMHTLLGVGS
Thunberg fritillary mosaic virus	**TFMV**	YP_254713.1	QMKAAALKNARTRMFGLDGSV	**GTTEED**	TERHTTTDVNRSMHSMLGIRM
Tobacco etch virus	**TEV**	ABJ16044.1	QMKAAAVRNSGTRLFGLDGNV	**GTAEED**	TERHTAHDVNRNMHTLLGVRQ
Tobacco vein mottling virus	**TVMV**	NP_056867.1	QMKAGRTPQFAAAMFCLDGSV	**SGQEEN**	TERHTVDDVNAQMHHLLGVKG
Turnip mosaic virus	**TuMV**	QBQ58063.1	QMKAAALRGANNNLFGLDGNV	**GTTVEN**	TERHTTEDVNRNMHNLLGVQG
Water melon mosaic virus	**WMV**	AXU24938.1	QMKAAALAGINSRLFGLDGNI	**STNSEN**	TERHTARDVNQNMHTLLGMGP
Wild melon vein banding virus	**WMVBV**	YP_009407950.1	QMKAAALRNANRRLFGIDGSV	**SSGEEN**	TERHTVEDVDRDMHTLLGMRK
Wild tomato mosaic virus	**WTMV**	QBB78857.1	QMKAAALRNANNRMFGLDGKV	**GTQEED**	TERHTAEDVNRNMHNLLGVRG
Yambean mosaic virus	**YBMV**	YP_004936165.1	QMKAAALSNVNNKLFGLDGNI	**ATTSEN**	TERHTARDVNQNMHTLLGMGP
Yellow mosaic virus	**YMV**	NP_612218.1	QMKAAAIGNKVNKMFGFDGKV	**GLPEED**	TERHAAGDVTKDMHSLLGMRG
Zucchini shoestring virus	**ZSSV**	YP_009665156.1	QMKAAAIRGANKRLFGIDGSV	**SEGGEN**	TERHTVEDVTRDMHSLLGMRN
Zucchini tigre mosaic virus	**ZTMV**	AGY36215.1	QMKAAALRNANRRLFGIDGSV	**SNREEN**	TERHTVEDVDRDMHSLLGMRK
Zucchini yellow mosaic virus	**ZYMV**	AWX33674.1	QMKAAALSNVSSRLFGLDGNV	**ATTSED**	TERHTARDVNRNMHTLLGVNT

Alignment of partial sequences of the coat protein (CP) from 79 potyvirus species. Aligned fragment spans from amino acids 282 to 329 of the CP of *Plum pox virus*. Casein kinase II (CK2) motif is highlighted with yellow background. Residues aligning with amino acid T243 of PVA CP are in red font. Amino acids numbering refers to the sequence of PPV-R. Proteins were aligned using the Clustal Omega software (*European Bioinformatics Institute*).

Supplementary Tables

Table S1

Table S1. Cloning of plasmids bearing mutations at casein kinase II motif of the coat protein of *Plum pox virus*

Construct [a]	***PCR step*** [b]	***Primers***	***Template***
**R-T304A**	PCR1	SM35-F-mut-Ala	pICPPV-NK-lGFP
		SM30-R-ext	
	PCR2	SM30-F-ext	
		SM35-R-mut-Ala	
	PCR3	SM30-F-ext	PCR1 + PCR2
		SM30-R-ex	
**R-T304D**	PCR1	SM36-F-mut-Asp	pICPPV-NK-lGFP
		SM30-R-ext	
	PCR2	SM30-F-ext	
		SM36-R-mut-Asp	
	PCR3	SM30-F-ext	PCR1 + PCR2
		SM30-R-ex	
**R-T304N**	PCR1	SM37-F-mut-Asn	pICPPV-NK-lGFP
		SM30-R-ext	
	PCR2	SM30-F-ext	
		SM37-R-mut-Asn	
	PCR3	SM30-F-ext	PCR1 + PCR2
		SM30-R-ex	
**SwCM-T306A**	PCR1	SM56-F-mut-T306A	pICPPV-SwCMCP-R
		SM30-R-ext	
	PCR2	SM59-F-ext	
		SM56-R-mut-T306A	
	PCR3	SM30-R-ext	PCR1 + PCR2
		SM59-F-ext	
**SwCM-T306D**	PCR1	SM57-F-mut-T306D	pICPPV-SwCMCP-R
		SM30-R-ext	
	PCR2	SM59-F-ext	
		SM57-R-mut-T306D	
	PCR3	SM30-R-ext	PCR1 + PCR2
		SM59-F-ext	
**SwCM-T306N**	PCR1	SM58-F-mut-T306N	pICPPV-SwCMCP-R
		SM30-R-ext	
	PCR2	SM59-F-ext	
		SM58-R-mut-T306N	
	PCR3	SM30-R-ext	PCR1 + PCR2
		SM59-F-ext	

[a] Mutated residues are in red.

[b] Mutations at T304 and T306 of PPV CP CK2 motif were introduced by site-directed mutagenesis using three-step PCR approach.

Table S2

Table S2. Primer list

Primer name	Sequence
Mutator primers for site-directed mutagenesis [a]	
**SM35-F-mut-Ala**	5′-TGGATGGAAACGTCGGA**GCA**CAAGAAGAGGACACAGAG-3′
**SM35-R-mut-Ala**	5′-CTCTGTGTCCTCTTCTTG**TGC**TCCGACGTTTCCATCCA-3′
**SM36-F-mut-Asp**	5′-TGGATGGAAACGTCGGA**GAT**CAAGAAGAGGACACAGAG-3′
**SM36-R-mut-Asp**	5′-CTCTGTGTCCTCTTCTTG**ATC**TCCGACGTTTCCATCCA-3′
**SM37-F-mut-Asn**	5′-TGGATGGAAACGTCGGA**AAT**CAAGAAGAGGACACAGAG-3′
**SM37-R-mut-Asn**	5′-CTCTGTGTCCTCTTCTTG**ATT**TCCGACGTTTCCATCCA-3′
**SM56-F-mut-T306A**	5′-TGGATGGAAACGTCGGA**GCA**CAAGAAGAGGACACAGAG-3′
**SM56-R-mut-T306A**	5′-CTCTGTGTCCTCTTCTTG**TGC**TCCGACGTTTCCATCCA-3′
**SM57-F-mut-T306D**	5′-TGGATGGAAACGTCGGA**GAT**CAAGAAGAGGACACAGAG-3′
**SM57-R-mut-T306D**	5′-CTCTGTGTCCTCTTCTTG**ATC**TCCGACGTTTCCATCCA-3′
**SM58-F-mut-T306N**	5′-TGGATGGAAACGTCGGA**AAT**CAAGAAGAGGACACAGAG-3′
**SM58-R-mut-T306N**	5′-CTCTGTGTCCTCTTCTTG**ATT**TCCGACGTTTCCATCCA-3′
Flanking primers for site-directed mutagenesis	
**SM30-F-ext**	5′-AGTCCTGCACAGGTTGACTTGTCAAACAC-3′
**SM30-R-ext**	5′-CGATTTAGGTGACACTATAGAATACAAGCTTCTAG-3′
**SM59-F-ext**	5′-GTTTGATCCCATATTCACTCCAGCAACAAC-3′
Primers for analysis of viral progenies	
**2429**	5′-GTCTCTTGCACAAGAACTAT-3′
**SM13-lGFP**	5′-TTACCTGTCCACAC-3′
**~~S~~80**	5′-TTGGGTTCTTGAACAAGC-3′

[a] Mutated codons in bold and underlined

Table S3

Table S3. Phospho-peptides identified in the coat protein of *Plum pox virus* virions purified from *Prunus persica* plants

Peptide sequence	Phosphorylation site	start-end	z	m/z	Search engine	Score
ADEREDEEEVDAGKPSVVTAPAA TSPILQPPPVIQPAPR[a]	S25[?]	1-39	4	1090.78	Mascot	>30
GNEDASPSNSNALVNTNRDR[b]	S81	76-95	3	737.65	Mascot	>40
DVDAGSVGTFTVPR[b]	S101	96-109	2	750.84	Mascot	>50
					Peaks	>50
AMTSKLSLPK[b]	S118	112-121	2	578.29	Mascot	>25
NLTDYSLAR[b]	T254	252-260	2	566.75	Mascot	>40
					Peaks	>50

Summary of confident peptides and their phosphorylated sites identified by LC-MS/MS, from two biological replicates. MS/MS spectra identifications were carried out by using Mascot and Peaks search engines, considering specified confident threshold scores, although manual validation was also performed. Data are available via ProteomeXchange with identifier PXD017780.

[a] Both post-translational modifications, phosphorylation and *O*-GlcNAcylation, are unambiguously detected in the identified peptide 1-39. However, y/b fragment ions assigning these modifications to specific residues could not be found. Phosphorylation at S25 (in green) is guessed on basis to previous results with virions purified from *Nicotiana benthamiana* plants, but phosphorylation in S16 or T19 cannot be ruled out. *O*-GlcNAcylation targeted one of these residues: S16, T24 or S25.

[b] Residues definitively found modified by phosphorylation are shown in red (see MS/MS fragmentation spectra in Supplementary Figure S2).

Table S4

Table S4. Summary of phosphorylations affecting the coat protein (CP) of different *Plum pox virus* isolates, and in different hosts

Plant host	Strain Isolate	Specific residues in PPV-R CP									
		pS25	pS62	pT71	pS81	pS101	pT106	pS118	pT254	pT304	pT313
Nicotiana spp.	D R	M	N.D.	M	M	M	M	M	M	N.D.	M
	Rec BOR-3	N.A.	M	N.D.	M	M	N.D.	M	M	N.D.	N.D.
	Cherry SwCM	-	N.A.	N.D. (S73)	M (pT83)	N.D. (T103)	N.D. (S108)	M (pS120)	M (pT256)	M (pT306	N.D. (T315)
Prunus persica	D R	?	-	N.D.	M	M	N.D.	M	M	N.D.	-

(M) phosphorylation mapped; (?) phosphorylation detected but not unambiguously assigned to that specific position; (N.D.) not detected phosphorylation; (N.A.) not applicable; (-) corresponding peptide not generated. Residues in the CP of the SwCM isolate in listed positions of R isolate are shown in brackets.

Table S5

Table S5. Infectivity of *Plum pox virus* mutants affected in putative phospho-target at the casein kinase II motif of the capsid protein (CP)

Inoculum [a]	Clone [a]	Infected / inoculated plants [b]
**R-T304A**	1	2/3
	2	2/3
**R-T304D**	1-1	3/3
	1-2	2/3
**R-T304N**	1	2/3
	2	2/3
**R**	1	3/3
	2	2/3
**SwCM-T306A**	1-1	4/4
	1-2	4/4
**SwCM-T306D**	1	2/4
	2	3/4
**SwCM-T306N**	1	4/4
	2	2/4
**CPSwCM-R**	1	4/4

[a] *Nicotiana benthamiana* plants were manually inoculated with the DNA of indicated plasmids. Two independent clones per construct were used, except in the case of mutants R-T304D and SwCM-T306A for which two DNA preparations from a single clone were used.

[b] Infectivity was estimated at 21 days post infection, based on CP detection by Western blot analysis and IC-RT-PCR amplification of the viral RNA.

References

1. Kim, Y. C.; Udeshi, N. D.; Balsbaugh, J. L.; Shabanowitz, J.; Hunt, D. F.; Olszewski, N. E., O-GlcNAcylation of the *Plum pox virus* capsid protein catalyzed by SECRET AGENT: characterization of O-GlcNAc sites by electron transfer dissociation mass spectrometry. *Amino Acids* **2011,** 40, (3), 869-76.

2. Pérez, J. J.; Udeshi, N. D.; Shabanowitz, J.; Ciordia, S.; Juárez, S.; Scott, C. L.; Olszewski, N. E.; Hunt, D. F.; García, J. A., *O*-GlcNAc modification of the coat protein of the potyvirus *Plum pox virus* enhances viral infection. *Virology* **2013,** 442, 122-131.

3. Martínez-Turiño, S.; Pérez, J. J.; Hervás, M.; Navajas, R.; Ciordia, S.; Udeshi, N. D.; Shabanowitz, J.; Hunt, D. F.; García, J. A., Phosphorylation coexists with *O*-GlcNAcylation in a plant virus protein and influences viral infection. *Mol. Plant Pathol.* **2018,** 19, (6), 1427-1443.

4. Hervás, M.; Navajas, R.; Chagoyen, M.; Garcia, J. A.; Martinez-Turiño, S., Phosphorylation-related cross-talk between distant regions of the core region of the coat protein contributes to virion assembly of P*lum pox virus*. *Mol. Plant Microbe Interact.* **2020**. In-press, doi: 10.1094/MPMI-10-19-0305-R.
